# The Role of Chimeric Antigen Receptor-T Cell Therapy in the Treatment of Hematological Malignancies: Advantages, Trials, and Tribulations, and the Road Ahead

**DOI:** 10.7759/cureus.13552

**Published:** 2021-02-25

**Authors:** Sai Rohit Reddy, Adiona Llukmani, Ayat Hashim, Dana R Haddad, Dutt S Patel, Farrukh Ahmad, Majdi Abu Sneineh, Domonick K Gordon

**Affiliations:** 1 Internal Medicine, California Institute of Behavioral Neurosciences & Psychology, Fairfield, USA; 2 Internal Medicine, Scarborough General Hospital, Scarborough, TTO

**Keywords:** adoptive immunotherapy, hematological malignancies, side effects of car therapies, car-t cells in hematological malignancies, car-t cell design, next generation car-t cells, immunotherapy and hematological malignancies, car-t cell therapy, safety strategies in car therapy, fda approved car therapies

## Abstract

Immunotherapy is the upcoming trend in cancer treatment. Traditional cancer treatment methods include surgical resection, radiotherapy, chemotherapy, small molecule targeted drugs, monoclonal antibodies, and hematopoietic stem cell transplantation (HSCT). Surgical resection is useful for early-stage patients but not for metastatic cancer cells; radiotherapy and chemotherapy are more common but produce substantial damage to normal tissues and have poor selectivity. Targeted drugs, including monoclonal antibodies, have better comprehensive efficacy but can also encourage gene mutation of tumor cells and drug tolerance. HSCT is effective, but choosing a donor is often difficult, and the graft is also prone to rejection. Thus, chimeric antigen receptor (CAR)-T cell therapy, a form of cellular/adoptive immunotherapy, is at the forefront of cancer therapy treatments due to its sustained remission, fewer side effects, and a better quality of life. CAR-T cell therapy involves genetically modifying the T cells and multiplying their numbers to kill cancer cells. This review article gives an insight into how the CAR-T cells have evolved from simple T cells with modest immune function to genetically engineered robust counterparts that brought great hope in the treatment of hematological malignancies. Much research has been undertaken during the past decade to design and deliver CAR-T cells. This has led to successful outcomes in leukemias, lymphomas, and multiple myeloma, paving the way for expanding CAR therapy. Despite tremendous progress, CAR-T cell therapies are faced with many challenges. Areas for improvement include limited T cell persistence, tumor escape, immunosuppressive components in the tumor microenvironment, cancer relapse rate, manufacturing time, and production cost. In this manuscript, we summarize the innovations in the design and delivery of CAR technologies, their applications in hematological malignancies, limitations to its widespread application, latest developments, and the future scope of research to counter the challenges and improve its effectiveness and persistence.

## Introduction and background

Immunotherapy is an upcoming trend in cancer treatment that aims to enhance the host immune system to recognize and destroy cancer cells. It includes monoclonal antibodies, immune checkpoint inhibitors, cancer vaccines, adoptive cell therapy, and cytokines. Adoptive immunotherapy is a type of immunotherapy that involves modifying the T cells, which act as "living drugs" that multiply in the patient's body, recognize the cancer cells that possess the specific antigen, and kill them [1]. Chimeric antigen receptor (CAR)-T cell is not a drug used in chemotherapy; instead, it is a living biologic manufactured from the patient's blood cells. This kind of cancer therapy leads to sustained remission, lesser side effects, and better quality of patients' lives, making it the preferred treatment plan over the other conventional cancer treatments like chemotherapy, radiotherapy, and surgical excision [2].

T cells play a vital role in the cell-mediated immune response. During the past few years, many therapies have been developed to redirect, culture, and enhance T cells against tumors [3]. The T cell-based adoptive immunotherapy revolves around developing new means to deal with malignancies, especially hematologic cancers. This emerging therapy includes three models: tumor-infiltrating lymphocytes (TILs), T cell receptor (TCR)-modified T cells, and CAR-T cells [2]. As compared to CAR-T cell therapy, the first two techniques do not make a considerable modification of the T cells per se, so the efficacy is not substantial. Also, the process of production, the low success rate, and the dependence upon vaccination limit their development [2-5]. As an up-and-coming therapeutic regimen, CAR-T cells have stood the test of time for the last three decades, and many of its products have now been approved by the United States Food and Drug Administration (FDA) [6,7].

Advantages

The advantage of CAR-T cell therapy is the short treatment time required for completion. It scores over the other traditional cancer treatments in long-term remission with a better quality of patient's life. As compared to stem cell transplants, which also involve aggressive chemotherapy, most patients have a rapid recovery with CAR-T cell therapy [8]. Unlike TILs and TCRs, which are dependent on Major histocompatibility complex (MHC) expression of target cells for antigen recognition, CAR-T cells recognize the specific antigens, independent of antigen processing by the target cells and MHC restrictions, thus, in turn, killing tumor cells effectively [3,9,10].

Process of CAR-T cell therapy

The basic process of CAR-T cell therapy involves apheresis, followed by leukapheresis, where we withdraw blood from the patient's body, and lymphocytes (white blood cells) are separated. It is then forwarded to the lab for genetic engineering, where genes are then inserted via the inactive virus/non-virus vector into T cells to produce chimeric antigen receptors (CARs) on their surface. These re-engineered CAR-T cells are multiplied in the lab for roughly two to three weeks, then frozen and sent for reinfusion [3,6,11,12]. Simultaneously, we administer conditioning chemotherapy to lower the WBC's of the patient and set the stage for infusion of thawed CAR-T cells. The CAR-T cells then help to kill the tumor cells via granzyme/perforin-mediated apoptosis [12,13].

Design of CAR-T cells

CAR-T cells are composed of extracellular, transmembrane, and intracellular domains and a flexible hinge that also adds to target recognition efficiency [11]. The extracellular domain has an antigen-binding domain, i.e., single-chain variable fragment (scFv) that recognizes tumor-associated antigens. The intracellular domain consists of the immunoreceptor tyrosine-based activation motif (ITAM) of the TCR complex CD3ζ chain, which activates the costimulatory signal [3].

**Figure 1:**
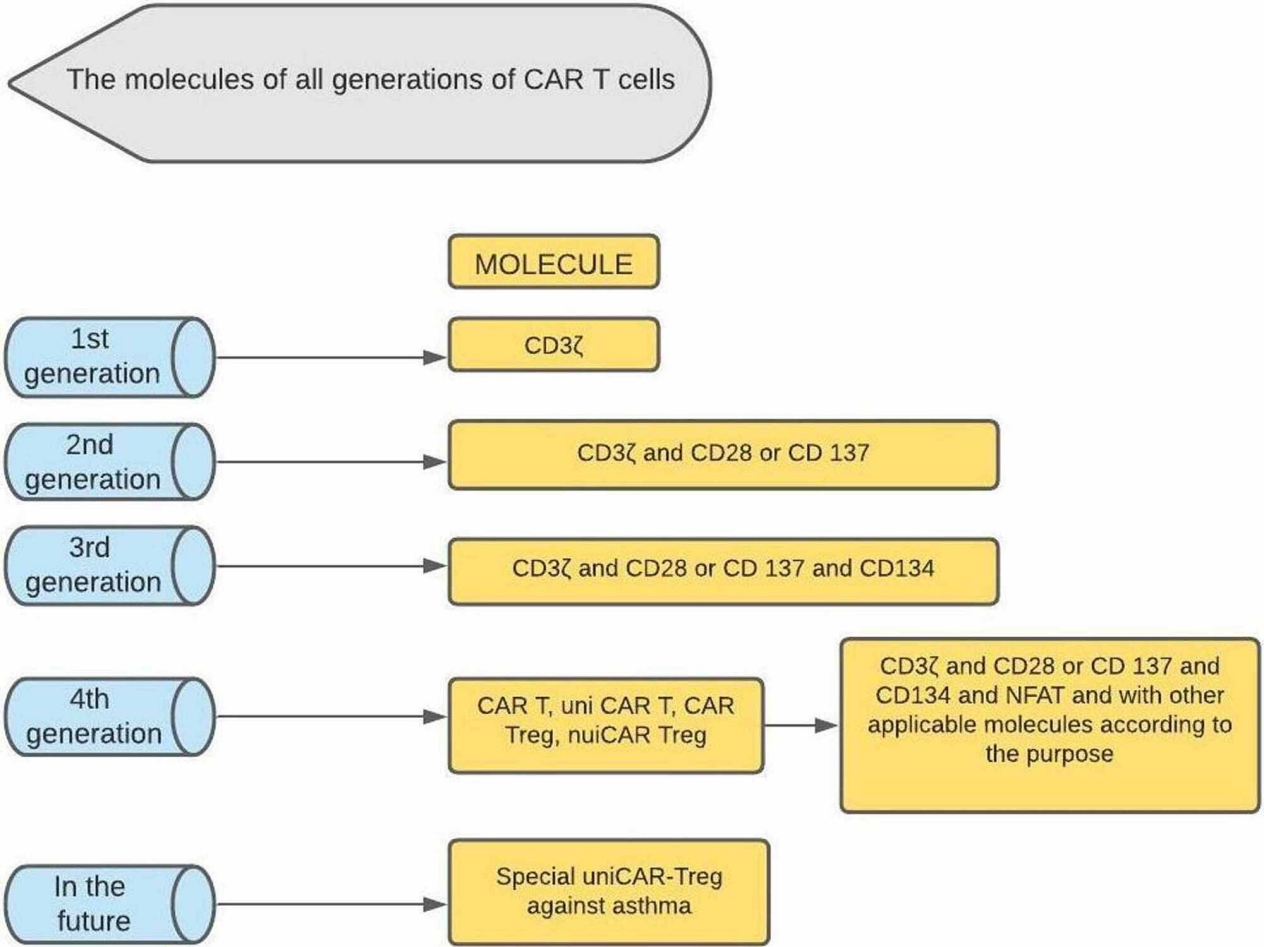
The different generations of CAR-T cells. (In addition to hematological malignancies, some fourth generation CAR T cells have also shown progress in the treatment of solid tumors, autoimmune diseases, and allergic diseases such as asthma. Antigen-specific Tregs and gene-edited T cell therapy have also shown encouraging results in controlling inflammation in allergic asthma.) CD: cluster of differentiation; UniCAR: universal CAR-T cells; Treg: regulatory T cells; NFAT: nuclear factor of activated T cells; CD3ζ: cluster of differentiation three zeta; CAR: chimeric antigen receptor

CAR technologies are being widely researched and developed into successive generations. The first-generation CARs contain a single-chain variable fragment (scFv), linkers, a transmembrane domain, and intracellular CD3ζ ITAMs [2,14,15]. The second-generation CARs have an additional intracellular costimulatory domain, e.g., CD28 or the 4-1BB receptor (CD 137). The third-generation CARs have two additional costimulatory domains, e.g., CD 28 or CD 4-1BB and CD 134. The fourth-generation CAR-T cells additionally consist of various costimulatory components such as cytokines, antibodies, or other functional proteins [14,15]. They can identify and remove some antigens that are not recognized by earlier generation T cells. A summary of different generations of modified and refined CAR-T cells has been enlisted below (Figure 1).

Application of CAR technologies in the treatment of hematological malignancies

To date, CAR-T cell therapy has been tried and tested in a variety of hematological malignancies, including relapsed/refractory (r/r) mantle cell lymphoma, B cell non-Hodgkin lymphoma-B-NHL (primary mediastinal B cell lymphoma, diffuse large cell lymphoma, and follicular lymphoma), multiple myeloma (MM), chronic lymphoblastic lymphocytic leukemia (CLL), adult and pediatric relapsed/refractory acute lymphoblastic leukemia (ALL) [7,16,17]. The CD19-specific CAR-T cells in the B-ALL patients can achieve an average complete remission rate of 70-94% [2,18].

Professionals have been studying the CAR-T cell therapies with many permutations and combinations with failures/relapses, refractory treatments, and a few positive strides accomplished to date. Moreover, several of them were approved by FDA, including Kymriah from Novartis, which is being used mainly for children and young adults with ALL and also in diffuse large B cell lymphoma (DLBCL); Yescarta from Gilead, for use in DLBCL; and Kite's Tecartus, accepted as the first CAR-T treatment for r/r mantle cell lymphoma [6,19-22].

Clinical trials revolving around hematological malignancies have shown that CAR-T cell therapy has helped bring remission in patients with multiple futile cancer treatments, which lasted for years [8]. Besides, it can also prevent their cancer from getting worse, helps them to live longer, and in a few cases, they can also benefit from treatments such as stem cell transplantation [14]. However, the limitation is the relapse, which may be mediated by antigen escape and the limited persistence of CAR-T cells [18].

Challenges in chimeric antigen receptor technologies

Despite the undeniable benefits of CAR therapies, there are still many side effects required to be kept under check with utmost scrutiny while administering it. Neurotoxicity and the cytokine release syndrome (CRS) are the commonly encountered side effects in most patients. Others include CAR-T cell-related encephalopathy syndrome, macrophage activation syndrome, hemophagocytic lymphohistiocytosis, graft-versus-host disease, on-target/off-tumor toxicity, anaphylaxis, and infections [3,8,23,24]. CRS occurs due to excess T cell proliferation and destruction of the cancer cells, causing an immune response in the body [3,16]. Neurotoxicity is a side effect in most patients with anti CD19 CAR-T cell therapy. Evidence of neurotoxicity includes endothelial dysfunction, vascular instability, capillary leak, blood-brain barrier disruption, and disseminated intravascular coagulation (DIC) [3,23,25]. As the same molecular biomarkers can be expressed in some normal tissues or organs, especially the lymphatic tissues, CAR-T cell therapy can destroy normal tissues referred to as on-target/off-tumor toxicity, e.g., B cell aplasia in anti-CD19/20 CAR-T cell treatment [3].

While there has been growing research in this area, the limited T cell persistence leading to relapse and adverse effects become bottlenecks to this approach's widespread use. Through this traditional review article, we intend to provide an insight into the advantages and successful outcomes of CAR-T cell therapy, the barriers for widespread adoption, and the expected modalities of ongoing research aiming to prevent resistance and relapses by not only focusing on the manufacturing of CAR-T cells but also by studying the effects of surrounding cells, previous treatments, tumor microenvironment causing CAR-T cells exhaustion and other relevant issues.

Search strategy and study selection

**Figure 2:**
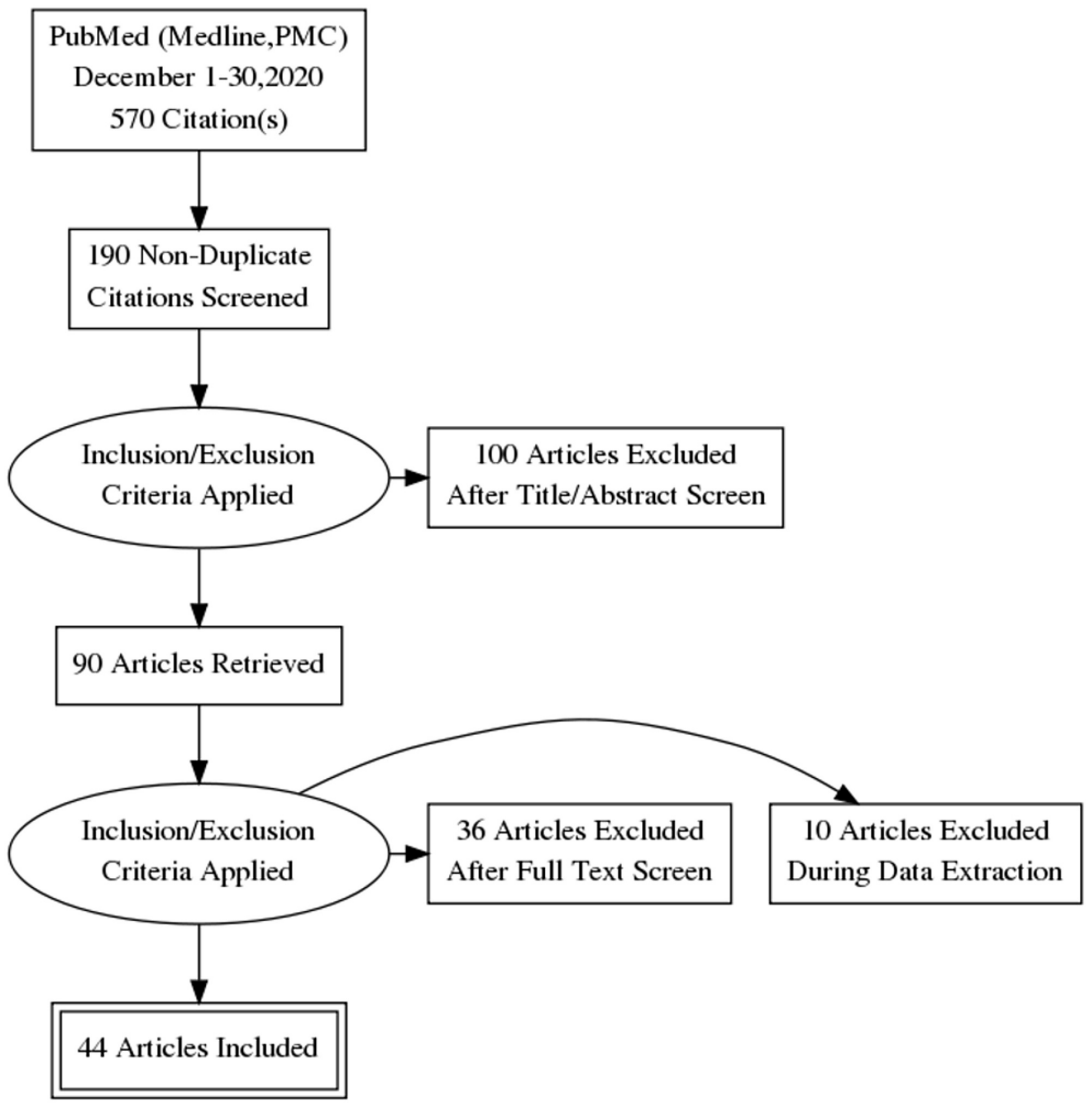
PRISMA flow diagram showing the data selection process. PRISMA: Preferred Reporting Items for Systematic Reviews and Meta-Analyses

During this review, we used PubMed and other electronic databases like Medline and PubMed Central to search for relevant publications. The keywords entered in the search process included "adoptive immunotherapy," "hematological malignancies," "immunotherapy and hematological malignancies," "CAR-T cell therapy," "next generation CAR-T cells," "approved CAR-T therapies," "side-effects of CAR therapies," "CAR-T cell design,' "safety strategies in CAR therapy," and "CAR-T cells in hematological malignancies." Articles were searched from January 2014 to November 2020 and later imported into Mendeley for organization and citations. After removing the duplicates, we reviewed all titles and abstracts using the inclusion and exclusion criteria. Figure 2 shows a summary of the study selection process in a Preferred Reporting Items for Systematic Reviews and Meta-Analyses (PRISMA) flow diagram.

Study selection criteria

All articles in the last six years (from January 2014), (2) articles in only English language, (3) studies originating from any country, and (4) all studies about adoptive immune therapy and CAR-T cell therapy in humans were included. In contrast, all publications before 2014, articles not available in English and not pertaining to humans, gray literature, and articles not available in full text were excluded. It was followed by reviewing the abstract for relevance, and then finally analyzing the full-text to meet our review article's goals.

## Review

**Figure 3:**
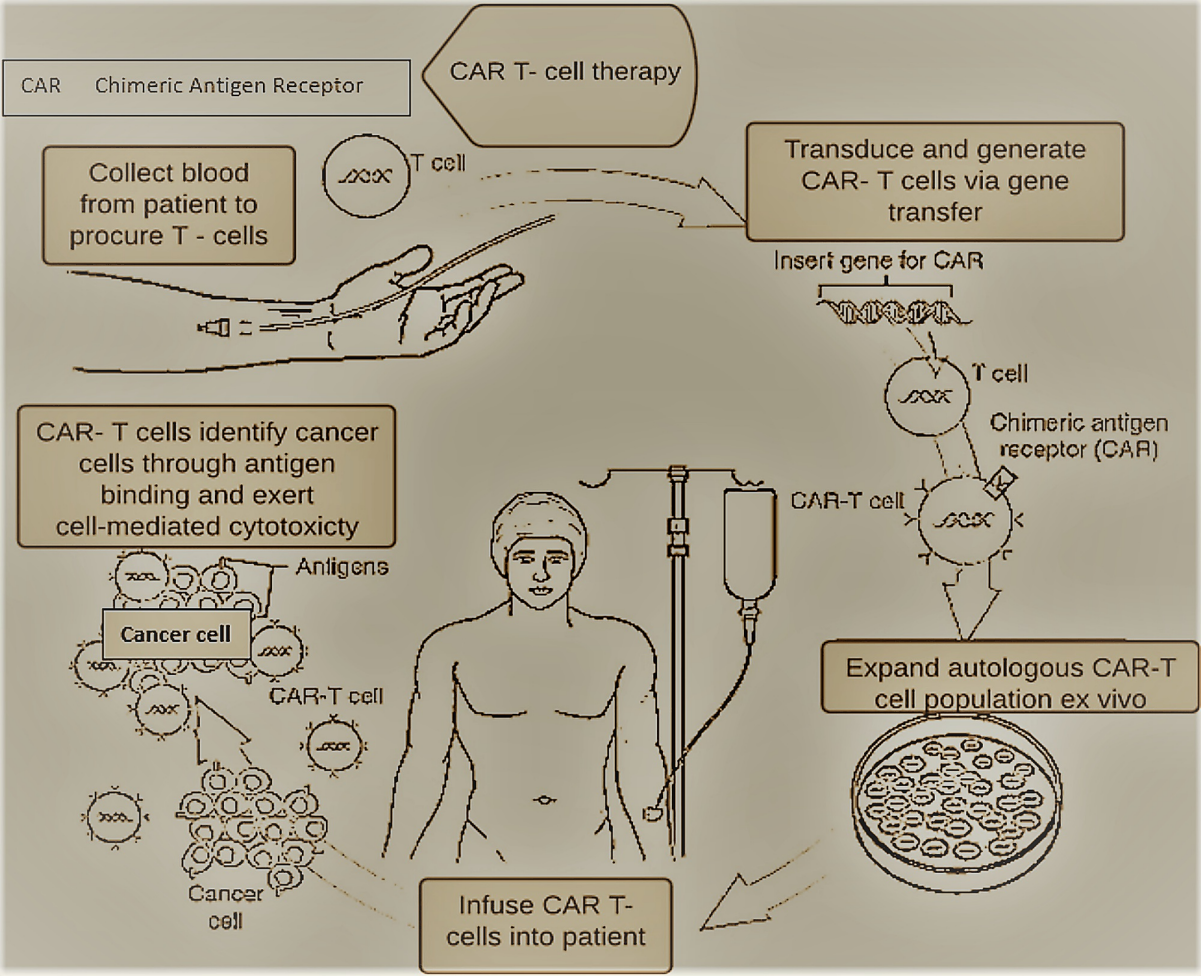
Overview of CAR-T cell treatment being administered to a patient. CAR: chimeric antigen receptor [3].

A novel breakthrough in cancer immunotherapy has been CAR-T cell therapy, with ongoing research spanning over 30 years. It activates the host's immune defenses rather than directly kill the tumor cells. Traditional cancer therapies like radiotherapy and chemotherapy have short-term therapeutic effects, cause side effects, and decrease patients' quality of life. In contrast, CAR-T cell therapy provides sustained remission and better quality-adjusted life years [2]. This therapy has been developing rapidly, with many globally established clinical trials with excellent commercial potential. The first study on this therapy was done by Gross et al. laying the groundwork for the first generation of CAR-T cells. There have also been advances in how long it takes to produce a batch of CAR-T cells as it initially took several weeks, but many labs have now reduced the time to less than seven days. We have summarized the process of manufacture and delivery of CAR-T cells to a patient in the diagram below (Figure 3).

CAR design and innovations in the development of CAR technologies

There has been much impressive research and development in CAR-T cell design and delivery to improve the efficiency, persistence, infiltration, and anti-apoptosis ability to treat tumor cells [3,14,15]. The basic structure of CAR-T cell has an extracellular domain consisting of a tumor-targeting/antigen-binding structure, i.e., an antibody derived single-chain variable fragment (scFVs); a hinge region; a transmembrane domain; and an intracellular signaling domain consisting of an activation motif with the CD3 zeta chain (T cell receptor complex), that provides signals for T cell activation leading to the production of toxic cytokines (e.g., IFNγ, TNFα) to attack tumor cells. Unlike typical T cell receptors, CARs are synthetic receptors binding to the target cells 'surface antigens, independent of antigen processing by the target cells and independent of MHC restrictions, thus preventing the immune escape of tumor cells [11,13,15,26].

There are four generations of CAR-T cells, created via continuous modifications and improvement of the effects of intracellular signaling domains [3,14,15]. The first generation consists of an scFv antigen-binding epitope and a single CD3ζ intracellular domain. Their anti-tumor action was limited in vivo, with the decrease in T cell proliferation and cytokine secretion, ultimately leading to T cells' apoptosis. The second generation of CAR-T cells incorporated an additional costimulatory molecule, i.e., CD28 or the 4-1BB receptor (CD137), which increased CAR-T cell proliferation, cytokine secretion, and secretion of the anti-apoptotic proteins and also delayed the antigen-induced cell death. Third-generation CAR-T cells have two additional costimulatory molecules, i.e., 4-1BB and CD28, which helped them produce a significant amount of toxic cytokines (IFN-γ) compared to first-generation or second-generation CAR-T cells that enhance the lysis of tumor cells. However, recent studies report that only the second-generation CAR-T cells can activate CD3ζ and have more robust signal transduction and anti-tumor effects than the third-generation CAR-T cells. The fourth generation of CAR-T cells, also known as T cells redirected for universal cytokine killing (TRUCKs), contains an activated T cell nuclear factor transcriptional counterpart that helps them secrete specific cytokines (interleukin-12). These cytokines modify the tumor microenvironment (one of the significant causes of resistance to CAR-T cells of earlier generations) and reconstitute the immune system by recruiting and activating other immune cells to induce an immune response, creating a hostile environment for tumor survival [13,27].

However, further modification of CAR-T cells by adding additional CAR parts, to form the next-generation CAR-T cells, has led to an improvement in their effectiveness and persistence [15,28]. Reconstructing the CAR part is also a new approach to antigen escape or coexistence of multiple tumor antigens in lymphoma, multiple myeloma, and other types of malignancies.

Application of CAR-T cell therapies in hematological malignancies

Genetically modified autologous T cells with redirected specificity to tumor antigens may combine advantages of cellular therapy (amplified response), antibody therapy (specificity), and vaccine therapy (memory activity). Chimeric antigen receptor-T cell therapy has been emerging cancer immunotherapy that has shown remarkable results, especially in hematological malignancies. The extensive exploratory work in the application of these therapies has led to successful outcomes (Table 1) in treating malignancies in children and adults such as recurrent B cell acute lymphoblastic leukemia, chronic lymphocytic leukemia, lymphomas including non-Hodgkin's lymphoma; r/r follicular lymphoma (FL); and diffuse large B cell lymphoma and multiple myeloma [3]. Clinical trials of CAR-T cell therapy mainly focused on CD19, CD20, CD22, CD30, CD38, BCMA, GPC3, and other famous antigen targets. In the United States, clinical trials targeting CD19 accounted for more than 40% of the CAR-T cell trials. Till August 2017, there were approximately 200 clinical trials involving CAR-T cells worldwide, and roughly about 65% of them involved hematological malignancies, of which 80% involved CD 19 CAR-T cells targeting B cell cancers [2]. Currently, CAR-T cell projects in clinical research involve more than 48 targets. As of June 2020, 357 clinical trials in China, 256 trials in the United States, and 58 trials in other countries have been registered [29]. 

**Table 1:** FDA-approved CAR-T cell immunotherapies/treatments in hematological malignancies. ALL: acute lymphoblastic leukemia; CAR: chimeric antigen receptor; CD: cluster of differentiation; CRS: cytokine release syndrome; DLBCL: diffuse large B-cell lymphoma; IL: interleukin; MCL: mantle cell lymphoma

Trade name/generic name manufacturer	Reference	Drug type	Treatment	Target	FDA approval
*Kymriah* (*Tisagenlecleucel*) - Novartis Pharmaceuticals Corporation	[20]	Immunotherapy	Pediatric and young adult patients with ALL and DLBCL	CD19 protein found on the surface of cancer cells	August 2017
*Yescarta *(*Axicabtagene ciloleucel*) - Kite Pharma, Inc	[21]	Immunotherapy	Adults with DLBCL	CD19 protein found on the surface of cancer cells	October 2017
*Tecartus* (*Brexucabtageneautoleucel*) - Kite Pharma, Inc (Gilead)	[22]	Immunotherapy	Relapsed or refractory MCL	CD19 protein found on the surface of cancer cells	July 2020
*Actemra* (*Tocilizumab*) - Genentech, Inc	[30]	Anti-cytokine therapy (monoclonal antibodies)	Adults and pediatric patients of 2 years of age or older with CAR-T cell-induced CRS	Interleukin-6 (IL-6) receptor	August 2017

Limitations in the widespread application of CAR-T cell therapies

Though there is much progress achieved, the usage of CAR-T cell therapy still involves many challenges.

Unfavorable Side-Effects

After CAR-T cell infusion, patients may encounter some severe side effects such as CRS and neurological toxicities, as the cells multiply in the body to fight cancer [3,6,25,31]. The extent of these side-effects (Table 2) and efficacy of CAR treatment depends upon the treatments that the patient has undergone previously, the amount of lymphodepletion, chemotherapy regimen, and the extent of disease at the time of infusion [28,32,33].

**Table 2:** Side-effects of CAR-T cell therapy and their treatments. CAR: chimeric antigen receptor; CD: cluster of differentiation; CRP: C-reactive protein; CRS: cytokine release syndrome; GVHD: graft-versus-host disease; HSCT: hematopoietic stem cell transplantation; ICANS: immune effector cell-associated neurotoxicity syndrome; IL: interleukin; IFN-γ: interferon-γ; MAS: macrophage activation syndrome; scFy: inhibitory chimeric antigen receptor; TCR: T cell receptor; TNF-α: tumor necrosis factor-alpha; TLS: tumor lysis syndrome

Side-effect	Treatment	Remarks	References
Cytokine release syndrome (CRS)/Cytokine storm: (a) cytokines are proteins that immune cells release when they attack an infection. The rapid and massive release of cytokines such as tumor necrosis factor-alpha (TNF-α), interleukin-6 (IL-6), and interferon-γ (IFN-γ) into the bloodstream of patients can lead to fever, chills, hypotension, tachycardia, trouble breathing, and low oxygen. (b) CRS is considered an "on-target" effect of CAR-T cell therapy—as its presence shows that the T cells are in action, releasing the cytokines that stimulate the immune response against tumor cells.	1. Monitor vital signs, Ferritin levels, C-reactive protein (CRP) level. 2. Low-grade CRS management involves supportive care, including antipyretics, evaluation for alternative sources of fever, intravenous fluids for hydration, and antiemetics' for nausea. 3. Higher-grade toxicities may require intravenous fluid boluses, support for hypotension, and high-flow oxygen for hypoxia. 4. Tocilizumab (Actemra), an Interleukin -6 receptor antagonist (IL-6 is a major cytokine induced by CAR therapy), is used as a first-line treatment for CRS. Systemic corticosteroids are used for patients with life-threatening CRS.	The rapid destruction of engineered T cells depresses the CD19 CAR- T cells' anti-tumor efficacy and may trigger subsequent disease progression or relapse.	[3,4,8,30, 34]
Immune Effector Cell-associated Neurotoxicity Syndrome (ICANS): the diverse range of neurological toxicities include symptoms such as headache, dizziness, encephalopathy, confusion, delirium, decreased consciousness, language impairment (aphasia), brain edema, and seizures.	1. The symptoms may resolve without much intervention in the majority of cases. 2. The standard care for ICANS includes supportive care and the administration of corticosteroids. Tocilizumab and intravenous corticosteroids are used in severe cases.		[3,4,35]
On-target, Off-tumor Toxicity: CARS usually attack tumor-specific antigens. Sometimes, tumor-specific antigens are also expressed on healthy cells in essential tissues such as the heart, lung, or liver, which may be attacked by CAR-T cells, posing life-threatening risks.	Immunoglobulin replacement therapy	While developing a CAR/TCR against an unrecognized target antigen on the tumor, its expression on normal tissues also needs to be examined.	[10]
B-Cell Aplasia: CAR-T cell therapy, which targets antigens present on the surface of B cells, destroys not only the cancerous B cells but also normal B cells (low numbers/ absence of B cells), leading to less availability of antibodies that protect against infection.	To prevent infections, Intravenous or subcutaneous immunoglobulin replacement therapy may be required.		[4,6]
Macrophage Activation Syndrome (MAS): in patients suffering from chronic autoimmune and rheumatic diseases, severe CRS is often associated with excessive multiplication of macrophages and T Cells.	Tocilizumab in mild cases and intravenous corticosteroids in severe cases		[6]
Tumor Lysis Syndrome (TLS): after aggressive cancer treatment, the dead cancer cells enter the bloodstream and disrupt the blood chemical balance, leading to organ damage.	Splitting the initial dose of CAR-T Cells administration, monitoring of vital parameters along with the administration of corticosteroids.		[36]
Anaphylaxis: a life-threatening allergic reaction may occur in patients after repeated T cell infusion due to IgE antibody response against the scFv component of the CAR itself. Symptoms include facial swelling, low blood pressure, hives, and respiratory distress.	Proper monitoring and immediate treatment of this life-threatening side effect are essential for patients receiving CAR-T cell therapy.		[6]
Graft versus host disease (GVHD): GVHD is a cause of concern in patients who received prior hematopoietic stem cell transplantation (HSCT). It is triggered by the reactivity of donor-derived / allogeneic CAR T cells against recipient tissues.	Controlled by administering glucocorticoids and monoclonal antibodies.		[37]

Factors That Affect the Potency of CAR-T Cell Activity

Other factors like immunogenicity (in case of repeated dosing, humanized scFvs should be used rather than mouse antibodies to prevent anaphylaxis or other severe dysfunctions), immunosuppressive components in the tumor microenvironment, immunosuppressive pretreatment prior to CAR-T infusion (causing anemia, coagulopathy, and neutropenic sepsis) may affect CAR-T cell activity and safety [10,32]. After the completion of treatment, the rate of cancer relapse is also a significant issue concerning CAR-T cell therapy's overall efficacy [18,38,39].

Latest developments to improve the safety and efficiency of CAR-T cell treatment, to counter the challenges in its application

Next-Generation CAR-T Cells

Researchers have been developing some safety strategies to prevent or reduce the adverse effects of CAR-T cell therapies. The T cells so manufactured are called next-generation CAR-T cells [9,28,31,40].

Suicide gene switch: It includes expressing a specific gene into the CAR T cells, which acts as a 'safety suicide gene switch' (activated by administering non-toxic molecules or associated monoclonal antibodies) to destroy the CAR T cells whenever there is a development of severe toxicity [9,31].

Synthetic splitting receptor: In synthetic splitting receptors proposed by Lim and colleagues, the timing, location, and dosage of T cell activity can be precisely and remotely controlled by pharmacologic regulation. In this system, the antigen binding and intracellular signaling components are separated in the absence of small molecules, and assembly starts after treatment with a heterodimerizing small molecule. Thus, the CAR-T cells can be effectively controlled with this small molecule in vivo, and the magnitude of responses such as target cell killing can be adjusted simply by changing the dosage of the small molecule. Another synthetic splitting receptor by Juillerat et al. is that the CAR architecture can be directly activated at the hinge domain with the addition of a small dimerizing molecule drug. The engineered CAR-T cell demonstrates a significant cell lysis activity after the use of the molecule inducer in a dose-dependent manner. The development of these novel controlled CAR-T cells opens innovative and attractive ways to control potentially adverse effects by improving the safety of CAR-T cell therapy in the clinic [9,28].

Combinatorial target-antigen recognition: It involves engineering the T Cells with two different CARs, which may be fully activated only after recognizing a set of different antigens expressed simultaneously on the tumor cells; e.g., the combination of CAR + Chimeric costimulatory receptor, Bispecific Tandem CAR. The normal cells that express only one of these antigens cant activate these T cells, thus significantly reducing on-target/off-tumor toxicity [9,28].

Synthetic notch receptors: The recent development of synthetic Notch (synNotch) receptors has helped avoid native T cells, thus overcoming toxicity in immunotherapy. SynNotch is composed of an extracellular antigen recognition domain (usually a single-chain variable fragment, scFv), a Notch core regulatory region, and an intracellular domain (ICD) [9,28,40]. SynNotch receptors use an extracellular domain to recognize a target antigen. However, as seen in CARs, binding of the target antigen does not trigger T cell activation. A synNotch receptor for one antigen drives the subsequent expression of a CAR targeting a second antigen. Only when both antigens are present, T cells can be activated. T cells with these receptors improve their effectiveness and safety profile [9,28].

Bispecific T cell engager (BITE antibody composed of two single-chain antibodies): Bispecific T cell engagers are built by connecting two scFvs through a linker (connector), which can simultaneously bind to two specific antibodies (scFvs) [9,28]. One of the scFvs binds to T cells via the CD3 receptor, and the other to a tumor cell via a tumor-specific antigen. In the absence of small bispecific molecules, these CAR-T cells are inactive against normal tissues. These cells improve T cells' potency and tumor selectivity, thereby enhancing their efficacy and safety [9,28]. A constructed monoclonal antibody, Blinatumomab, which targets CD19, was approved in 2014 for relapsed and refractory B cell ALL. They are safe and effective in improving long-term outcomes when used, along with tyrosine kinase inhibitors [31,41].

Inhibitory chimeric antigen receptor: The application of inhibitory receptors to CAR-T cells acts as a safety strategy. The Inhibitory chimeric antigen receptor (iCAR) consists of an scFv specific to the antigens expressed on normal tissue and a robust inhibitory signaling domain (from PD-1 and CTLA-4) to restrict T cell activity outside the tumor. The iCAR shows antigen-specific suppression of T cell activity, cytokine secretion, and proliferation in a temporary and reversible manner [9,28,40]. 

Combination Therapies

Researchers have tried the combination of CAR-T cell therapy with chemotherapy and radiotherapy to overcome the restrictions on usage of CAR-T cell therapy, reduce the adverse effects of the disease, enhance tumor antigen recognition, and increase the efficacy and persistence of CAR-T cells [2,41]. The combination of CAR-T cells and checkpoint inhibitor therapy (target PD-1 and PD-L1, CTLA-4, and B7 interactions) can also address the issue of antigen escape [42].

Other Areas Explored

**Figure 4:**
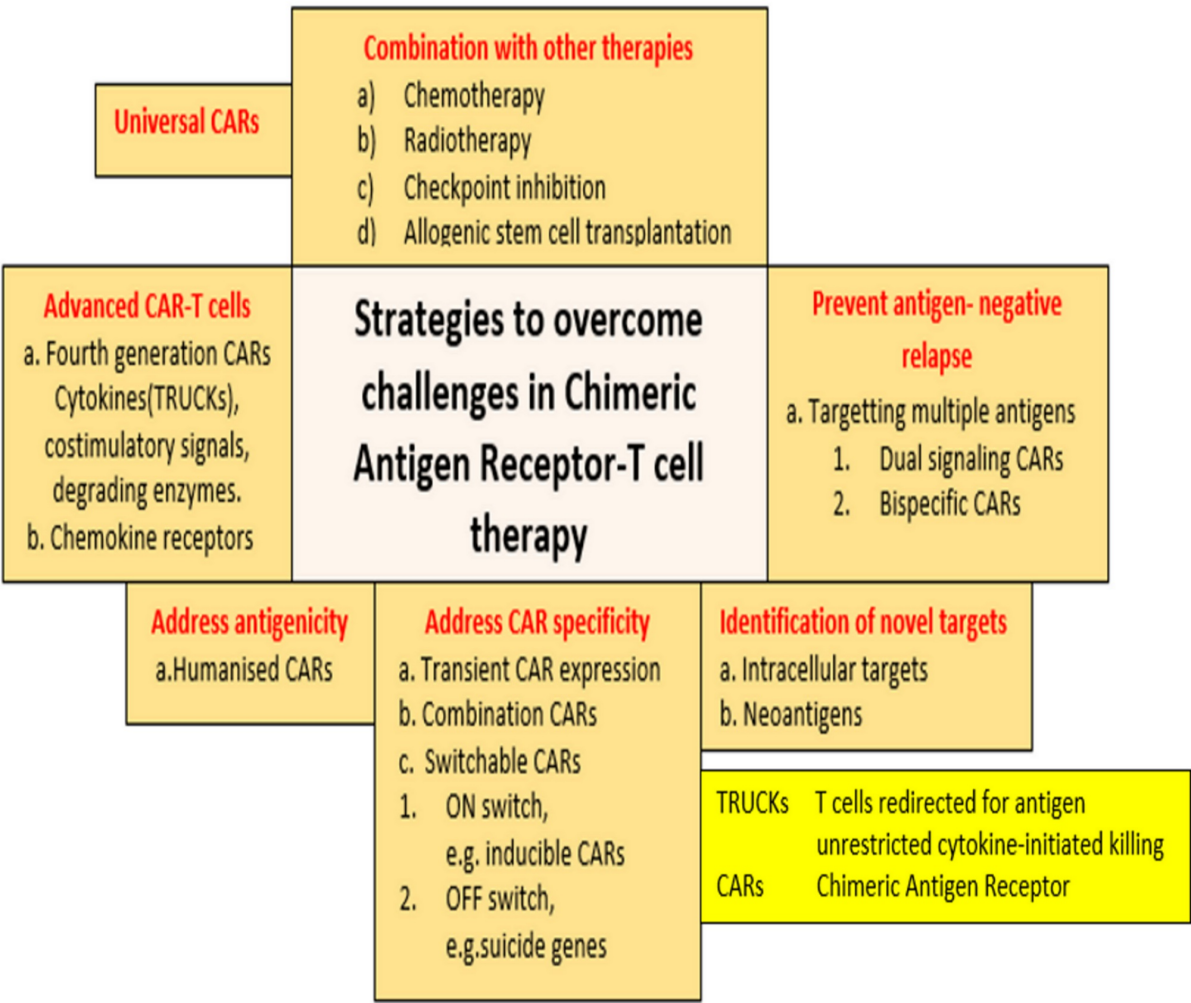
Summary of some of the strategies to overcome challenges in CAR-T cell therapy. TRUCKs: T cells redirected for antigen unrestricted cytokine‐initiated killing; CARs: chimeric antigen receptor

Other new areas of research in progress to improve the ability of CAR-T cells, resist the TME and decrease the immunogenicity [3,43] include (1) multitargeted CAR-T cell technologies; (2) Reforms in the manufacture of T cells (e.g., decrease the risks associated with using virus vectors, including DNA damage, and reduce cost and time of production: by replacing them with nonvirus-based technologies, such as Sleeping Beauty and PiggyBac transposition leading to the production of T cells with high persistence and low DNA damage [38,43], thus solving the various potential insertion-related mutations); (3) gene-editing techniques including the use of a nonintegrated lentiviral vector, recruiting CAR-T cells by oncolytic virus equipped with chemokine genes, generation of CAR-T cells with CRISPR/cas9 technology (clustered regularly interspaced short palindromic repeats/CRISPR- associated protein nine) and the use of CAR-NK (Natural killer) cells [2,44]. Apart from the above-mentioned technical aspects, one more area that one needs to address to make CAR therapy more accessible is the huge cost involved. At present, in the USA, the price of Tiga-Cel (Kymriah) is $475,000, and that Axi-Cel (Yescarta) is $350,000, which may not be affordable to most people in the world, which is limiting the use of this therapy [39]. We tabulated the various possible solutions to overcome the limitations of CAR-T therapy in order to aid in comprehension and a faster transition to application in the clinical field (Figure 4).

The success of ELIANA and ZUMA trials, resulting in FDA approval of several CAR-T cell therapies has led to progressive research related to the fourth generation and next-generation CAR-T cells, including the newest technologies, which are already under various phases of clinical trials around the world [45,46]. The long-term benefits will be established only after substantial positive results on a large scale. Therefore, we finally infer that innovative bench research is required to strengthen the effectiveness of the advances in CAR-T cell therapy that will give physicians and patients the information and therapeutics to eliminate the malignancies. This review article's limitations include a lack of statistical analysis among different studies in humans to compare individual effectiveness in various hematological cancers. Only studies over the past six years have been taken into consideration by the authors.

## Conclusions

Globally, researchers are constantly on the lookout for novel strategies to combat cancer. CAR-T cell immunotherapy is one such area, which is under close observation to bring about a revolution in cancer cure. The anti-tumor functioning of CAR-T cells has inspired many during the last decade to carry out various clinical trials and study the development, functions, and outcomes of CAR therapies. The FDA approval of some of the CAR treatments and their commercial viability heralded a new era in CAR Technologies. However, the limitations like unfavorable side effects, limited T cell persistence, tumor escape, immunosuppressive components in the tumor microenvironment (TME), rate of cancer relapse, and the huge cost of manufacturing involved are limiting the efforts of many to reap the full potential of CAR therapies. While the effectiveness of these inventions is being explored and established, it leaves a lot of scope and opportunities for future research to develop innovations in CAR technologies that can efficiently address the current limitations and lead to widespread adaptation of the treatment. The areas of interest for future researchers can be the optimization of CAR-T cell design, use of combined adoptive cell therapy/combination therapies, microenvironmental modification, and immune blockade strategies. Exploratory research to reduce relapse rates after achieving CR (complete remission) through multitargeted approaches or combination with other treatment methods is another critical area. More clinical studies may also be required to extend its application to cancers in other areas such as myeloid leukemia and solid cancers. In this review, we outlined the current status of CAR-T cell therapy growth, its applications in hematological malignancies, limitations, the latest developments in research to counter the challenges, and the future research scope to promote and extend its applications to existing and various other cancer treatments. Through this review, we intend to provide references for students, researchers, and colleagues in the field to better understand the present and future trends in CAR-T cell therapies.
